# Changes in paranasal sinus volumes, temporal bone pneumatization, internal acoustic canal and olfactory cleft dimensions over the centuries: a comparison of skulls from different epochs in Anatolia

**DOI:** 10.1007/s00405-024-08804-9

**Published:** 2024-07-08

**Authors:** Levent Yücel, Fatemeh Azizi, Salih Cengiz Meral, Çilem Sönmez Sözer, Ayla Sevim Erol, Zafer Ünsal Çoşkun, Timur Gültekin, Ceren Karaçaylı, Bülent Satar

**Affiliations:** 1 Department of Otorhinolaryngology, University of Health Sciences, Gülhane Training and Research Hospital, General Dr. Tevfik Sağlam Street, No:1, Etlik/Ankara, 06010 Turkey; 2 Department of Radiology, University of Health Sciences, Sultan Abdulhamithan Training and Research Hospital, Istanbul, Turkey; 3 Department of Anthropology, Faculty of Humanities, Ankara University, Ankara, Turkey

**Keywords:** Temporal bone pneumatization, Ancient skulls, Paranasal sinus volume, Olfaction, Hearing

## Abstract

**Objectives:**

Investigating changes in temporal bone pneumatization (TBP) and paranasal sinus volumes (PSV) across different eras may help understanding not only changes in skull anatomy but also pathophysiology of chronic otitis media and sinusitis, respectively, which are common health problems.

**Methods:**

Eight skulls from the second century AD, 20 skulls were from the 10th–11th centuries AD, 20 skulls from the 16th–19th centuries AD, and 60 contemporary skulls were included in this cross-sectional observational study. Using computerized tomography (CT) scans, the PSV were calculated by multiplying the height, width, and antero-posterior distance of the sinuses. TBP was divided into three types. Internal acoustic canal (IAC) length and width, and olfactory cleft (OC) width were measured.

**Results:**

No statistically significant differences were found between the paranasal sinus (frontal, maxillary, and sphenoid) volumes between the groups. However, TBP decreased statistically significantly over time on both sides of the skulls (*p* = 0.001). The contemporary IAC and OC measures were found to be significantly lower on both sides compared to the skulls from the other three eras (*p* < 0.001 for both).

**Conclusions:**

Although no significant change was observed in PSV, decreases were evident in TBP, OC width and IAC length and width over time. It appears a fair inference that changes in size of OC and IAC might be another indication of the fact that olfaction and hearing were more vital for survival in old eras. Since we do not know incidence of chronic ear problems in old eras, we cannot speculate outcome of increased TBP in terms of developing chronic ear diseases. On the contrary, increased TBP was likely to play a protective role in traumas in old ears. Additionally, the environmental influences may be crucial role in the development of paranasal sinuses.

**Supplementary Information:**

The online version contains supplementary material available at 10.1007/s00405-024-08804-9.

## Introduction

Throughout history, attempts have been made to understand the socioeconomic, cultural, and structural characteristics of societies that lived in previous ages. The study of human evolutionary processes, which have been progressive and gradual, can provide clinicians and researchers with unexpected and new perspectives on clinical problems. Such evolutionary developments are necessarily the result of a series of long-lasting adaptive processes, including anatomical changes [1]. The mummification of bodies in ancient Egyptian society for various spiritual or religious reasons, which has led to their greater preservation, has made mummies the focus of attention in numerous studies [2]. While skeletal remains from ancient times were first evaluated via macroscopic measurements, a new era began with the discovery of X-rays, and the first report containing radiographic imaging of mummies was published just one year following their discovery [3]. This clearly reflects people’s curiosity about past populations, their structural features, and the changes in humans. Similarly, with the discovery of tomography in the 1970s, paleoradiology began to be widely used [4], and Ludlow et al. [5] showed that computerized tomography (CT) provides more accurate results than conventional radiography.

Pneumatization is the infiltration of the epithelium into developing bone and the formation of air cell spaces lined with epithelia [6, 7]. The temporal bone is thus shaped with air-filled spaces. Aside from genetic conditions, temporal bone pneumatization (TBP) may be affected by prolonged otitis media, nutrition, the environment, age, and disease [8]. Chronic otitis media with effusion is a common health problem, and most researchers agree that small mastoid air cell systems correlate with long-standing otitis media [9]. Environmental factors such as increasing population sizes, exposure to smoking, and allergies are the other main causes of chronic otitis media with effusion, which is closely related to a decrease in TBP.

TBP has been accepted as an important indicator of the phylogenetic character of human evolution [10]. Accordingly, it is frequently used to determine taxonomic similarities [11, 12]. Most contemporary studies on TBP have consisted of clinical studies, as pneumatization is closely related to temporal bone diseases, such as chronic otitis media and cholesteatoma, and TBP is an important factor affecting surgical success and complications [13].

Various structural and physiological functions have been proposed for the paranasal sinuses over the centuries. These include the heating and humidification of inspired air, contributions to heat dissipation and balance, the addition of sound resonance, the production and/or storage of nitric oxide, biomechanical support, and a reduction in chewing stress [14]. Although many studies on paranasal sinus volumes (PSV) in ancient societies have been published in the literature, most of them have focused on gender estimation [15–17]. Marquez and Laitman [18] suggested that the paranasal sinuses may be affected by environmental conditions, and respiratory adaptation may take place over time. However, few studies have investigated PSV changes across different centuries [19]. Determining and understanding these changes may help solve the pathophysiology of chronic sinusitis, which is one of the more common health problems globally.

Olfaction and hearing are critical senses for life, but they were more important in ancient societies to protect people from dangerous situations. As far as we know, no researchers have examined the differences in the measurements of the internal acoustic canal (IAC) and olfactory cleft (OC) across different eras. Our hypothesis is that TBP, PSV, IAC and OC measurements have differed over the centuries. We therefore aimed to investigate the changing status of TBP, PSV, and IAC and OC measurements over the centuries. To our best knowledge, this is the first study in the English literature to compare them across different eras.

## Methods

### Study design

We designed a cross-sectional observational study to investigate the changing status of TBP, PSV, IAC and OC dimensions over the centuries. The manuscript was designed according to STROBE (Strengthening the reporting of observational studies in epidemiology) guideline.

### Participants

The ancient skulls used in this study were obtained from the Department of Anthropology, Faculty of Language, History and Geography, at Ankara University. Those with a macroscopically preserved appearance were selected and included. Fragmented or damaged skulls were excluded from the study. To compare all measurements between different centuries, skulls from four different centuries were included in the study.

### Groups

1. Cyzicus (Balıkesir, Türkiye): 2nd century AD.
2. Belentepe (Muğla, Türkiye): 10th − 11th centuries AD.
3. Karacaahmet Cemetery (Istanbul, Türkiye): 16th − 19th centuries.
4. Contemporary (Istanbul, Türkiye).

CT scans of 60 contemporary individuals from the archives of the PACS (picture archiving and communication system) were randomly selected, and 29 scans were of women. No pathologies were detected in either the temporal bones or paranasal sinuses. Skulls with temporal bone or paranasal sinus pathology were excluded.

### Features of the skulls

The materials belonging to the ancient city of Cyzicus have been dated to the second century AD, and the skeletal remains of four females and eight males found in a sarcophagus in the West Necropolis area to the northwest of the city during excavations in Balıkesir Province, Erdek District, have been dated to the same period. The skeletal remains of one female, three males, and one unidentified individual were recovered from a chamber tomb in the same area that belonged to 17 adult individuals. Among these materials, a total of eight adult skulls (three females and five males) were selected and included in this study.

The materials belonging to the Belentepe rescue excavations have been dated to the 10th–11th centuries AD. They consist of 188 individuals identified from 150 graves unearthed from the Necropolis area of the Eastern Roman period during excavations in the Milas District of Muğla Province. These 188 individuals comprise seven fetuses, 28 infants, 44 children, two adolescents, 33 women, 57 men, and 16 individuals whose gender and/or age could not be determined. The skulls of 20 adult individuals (10 females and 10 males) were selected and included in this study.

The materials obtained from the Karacaahmet Cemetery in Istanbul, which date back to the 16th–19th centuries, consist of the skulls of 20 adult individuals: four females and 16 males. All of them were included in the study.

### Imaging using CT

CT scans were obtained using a Canon Aquilion Lightning device (Toshiba Medical Systems, Japan) for the contemporary and ancient skulls, with 1 mm thickness, 200 mA, 120 kV, a 1 s rotation time, and 1 mm interval scanning parameters in the axial, coronal, and sagittal planes. The CT scans of the skulls were numbered, and the axial, coronal, and sagittal images were exported in a DICOM (Digital Imaging and Communications in Medicine) file format.

### Measurements

All constructions and measurements were performed on a 21.3-inch flat-panel colour active matrix TFT medical display (NEC MultiSync MD215MG, Munchen, Germany) with a resolution of 2048 × 2560 at 75 Hz and 0.17-mm dot pitch operated at 11.9 bits. The examiner was also permitted to use enhancements and orientation tools such as magnification, brightness, and contrast to improve visualization.

**Fig. 1:**
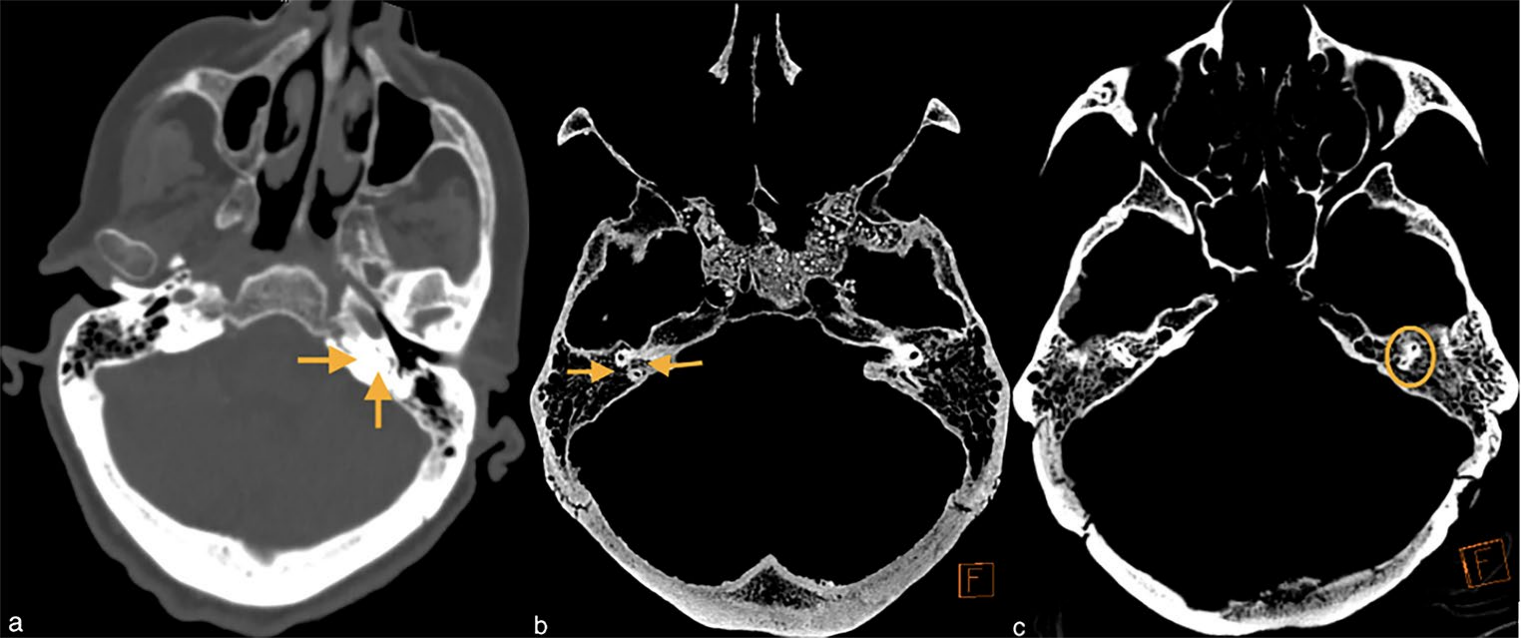
Types of temporal bone pneumatization (**a.** type 1: arrows indicate no pneumatization in peri-labyrinthine area [from contemporary skull], **b.** type 2: arrows indicate pneumatization medial and lateral to superior semicircular canal, **c**. type 3: circle indicates full pneumatization around superior semicircular canal)

Using the axial tomography sections, the TBP images were divided into three types in line with the work of Jadhav et al [6]. The pneumatization around superior semicircular canal (SSC) was the main criteria in this classification. The skulls without pneumatization around SSC were allocated to type 1 (Fig. 1a). Type 2 was defined as pneumatization both medial and lateral to SSC but not all peri-labyrinthine area (Fig. 1b), and type 3 was described as full pneumatization of the peri-labyrinthine area around SSC (Fig. 1c). These were measured separately for the right and left ears.

**Fig. 2:**
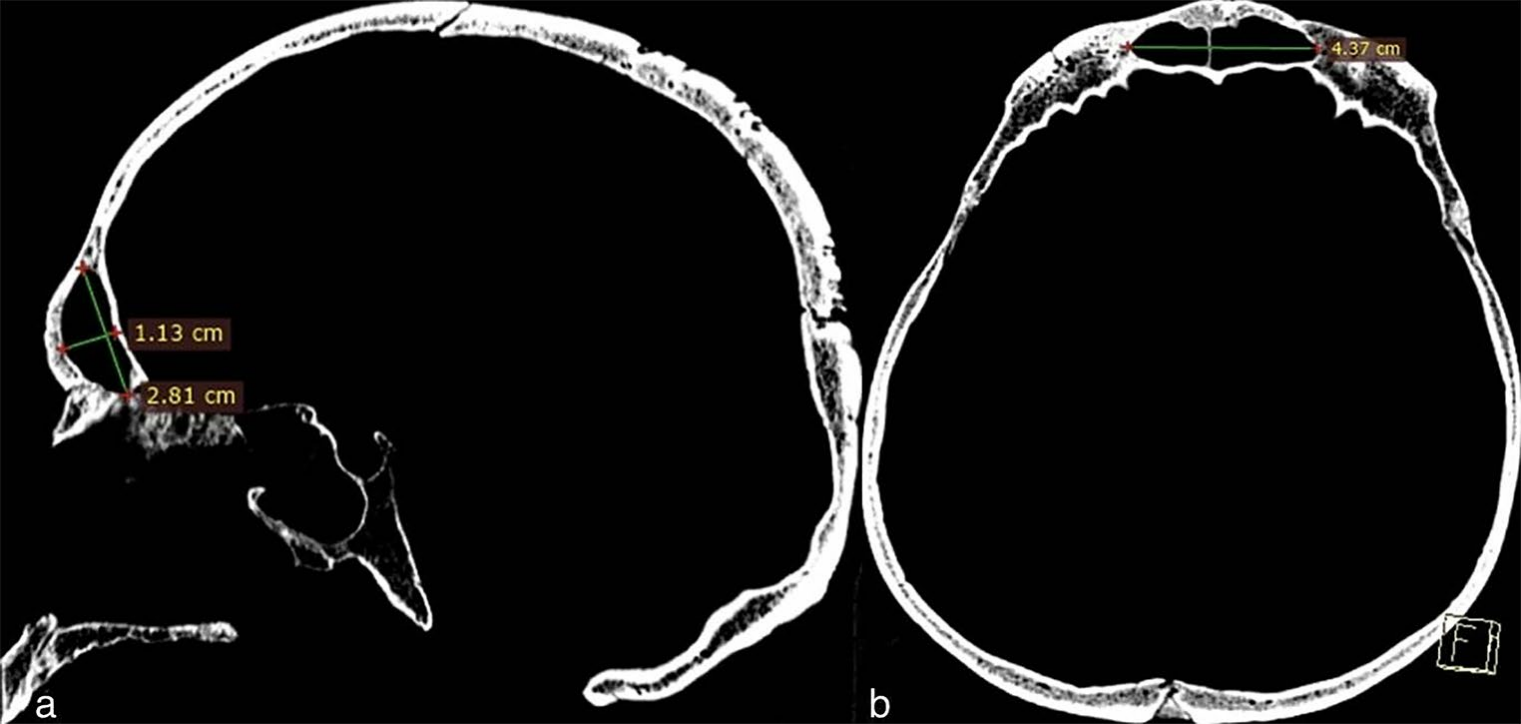
Measurement of frontal sinus volume

**Fig. 3:**
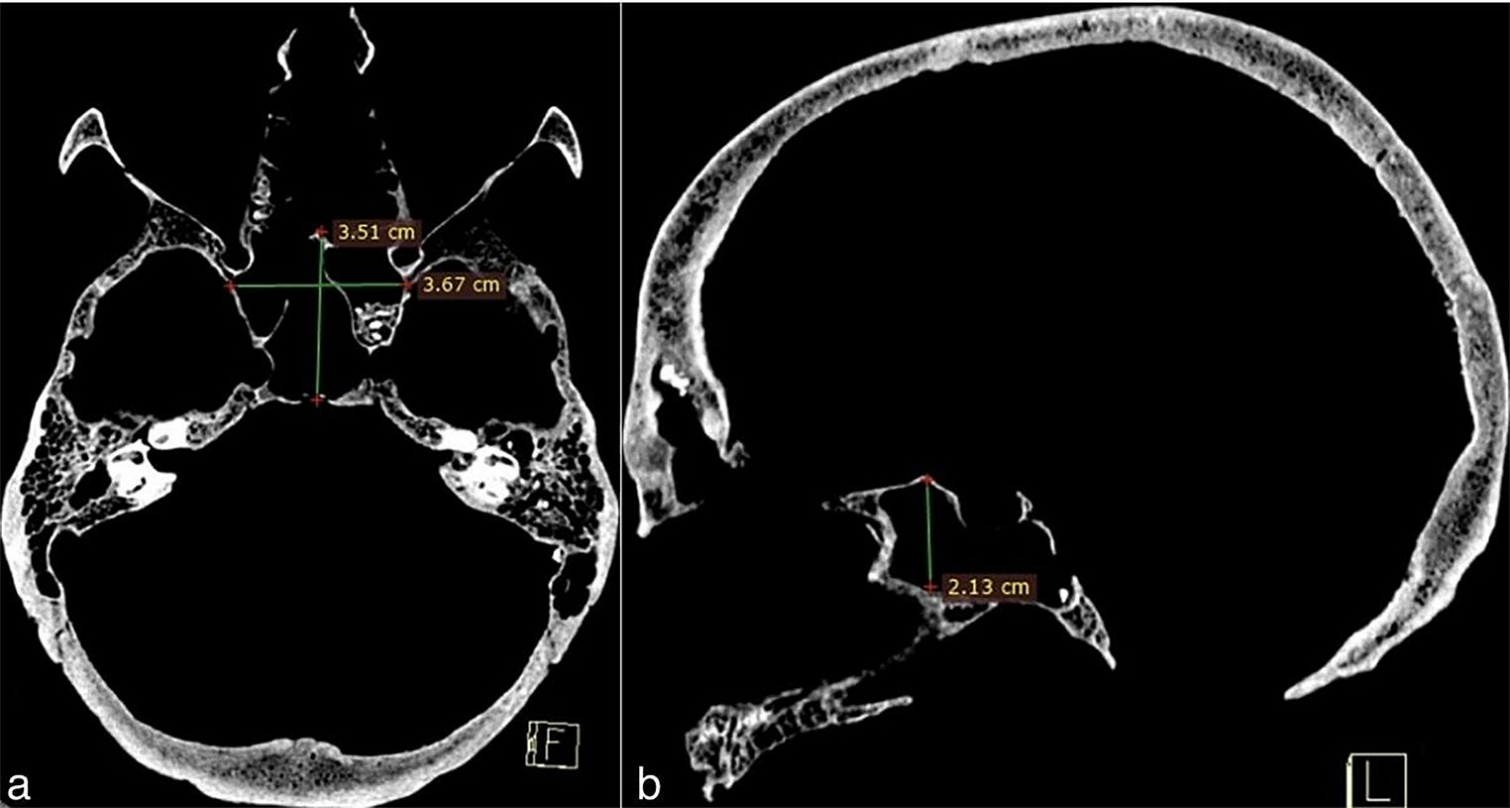
Measurement of sphenoid sinus volume

**Fig. 4:**
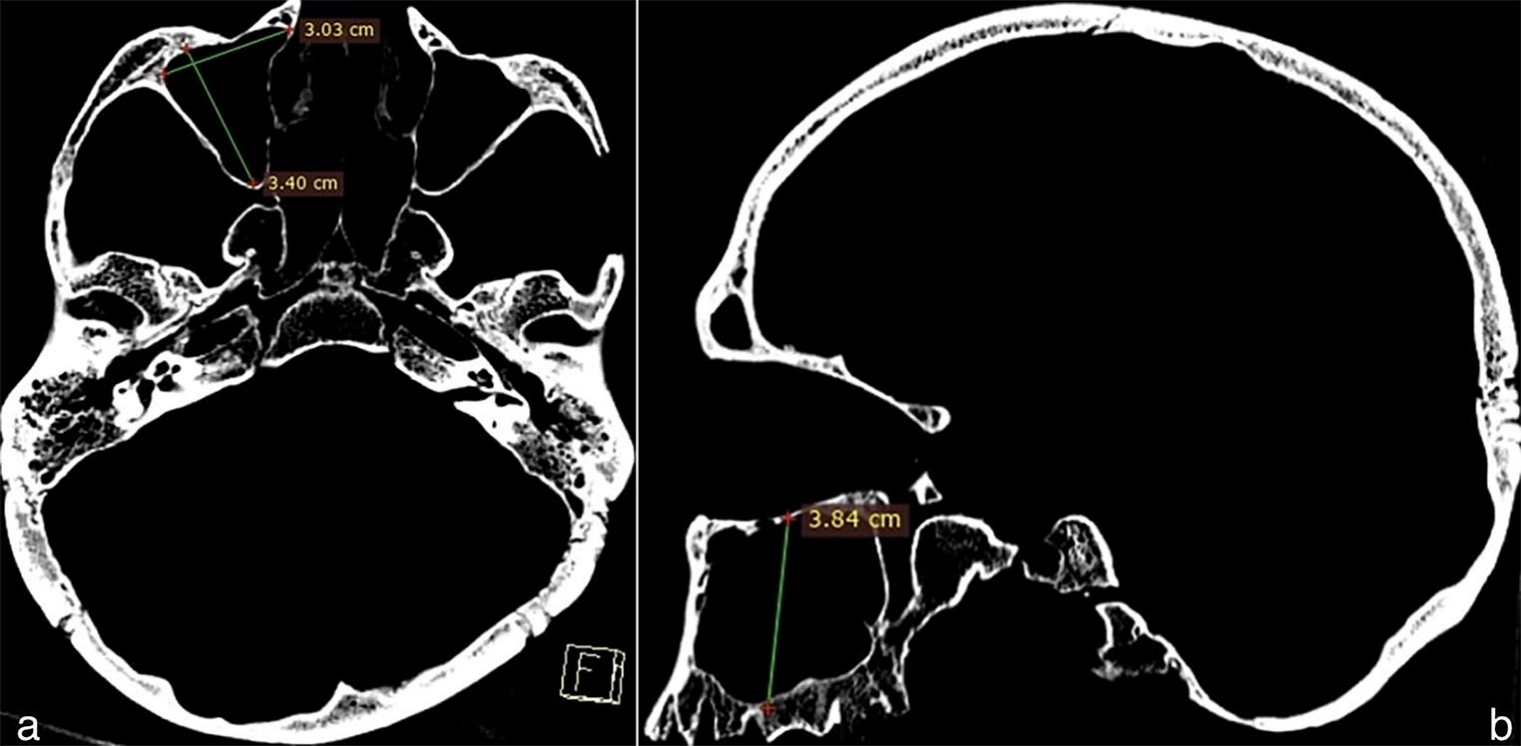
Measurement of maxillary sinus volume

The PSV (frontal, sphenoid, and maxillary) were obtained by multiplying the height, width, and antero-posterior distance from the axial, coronal, and sagittal sections, and the results were recorded in cubic centimeters (Figs. 2 and 3, and 4).

Using the coronal sections of the CT images, we also measured the IAC length and width (supplementary Fig. 1) and the OC width (supplementary Fig. 2).

### Missing data

Due to damage to the bone structures, the frontal, maxillary, and sphenoid sinus volumes could not be measured in one skull, and the frontal sinus volumes could not be measured in four other skulls. The IAC width and length could not be determined on the left in one skull, and the OC width could not be measured in five skulls due to damage to that area.

### Statistical analysis

The data were analyzed using RStudio version 3.6.3 (2020-02-29) “Holding the Windsock.” The descriptive statistics were presented as mean ± standard deviation (SD) and median (minimum–maximum) for the numerical variables and the number of cases (%) for the categorical variables. The normality hypotheses were tested with the Shapiro–Wilks test. Comparisons of the continuous variables between multiple groups were undertaken using ANOVA (using Welch’s correction, if necessary) or the Kruskal–Wallis test. Pairwise group comparisons of the continuous variables were evaluated with the independent samples *t*-test or the Mann–Whitney *U* test. The categorical data were compared using Fisher’s exact test. A result of *p* < 0·05 was considered statistically significant.

### Role of the funding source

The funder of this study had no role in study design, data collection, data management, data analysis, data interpretation, writing of the report, or the decision to submit for publication.

## Results

In this study, the remains of eight adults, three of whom were females and five males, were obtained from the Cyzicus Ancient City excavations and dated to the second century AD. Of the 20 skulls obtained from the Belentepe rescue excavations dated to the 10th–11th centuries AD, 10 were females. A further four females and 16 males’ skulls dated to the 16th − 19th centuries AD were obtained from the Istanbul Karacaahmet Cemetery study, and the contemporary tomography images of 60 patients (29 females, 31 males) were included in this study.

### Temporal bone pneumatization

The right temporal aeration measurements showed that all the skulls obtained from Cyzicus and Belentepe had type 3 pneumatization. While six (30%) of the skulls obtained from Karacaahmet were allocated to type 2, 14 (70%) had type 3 pneumatization. Of the contemporary skulls, six (10%) were assigned to type 1. We observed that 22 (36.7%) of these skulls had type 2 pneumatization, and 32 (53.3%) had type 3 pneumatization (Table 1).

**Table 1:** Comparison of groups in terms of temporal pneumatization

	2nd Century AD (*n* = 8)	10th -11th Century AD (*n* = 20)	16th -19th Century AD (*n* = 20)	Contemporary (*n* = 60)	p*
**Right TBP**					
Type 1	0 (0%)^a^	0 (0%)^a^	0 (0%)^a^	6 (10%)^a^	**0.001**
Type 2	0 (0%)^ab^	0 (0%)^b^	6 (30%)^a^	22 (36.7%)^a^	
Type 3	8 (100%)^ab^	20 (100%)^b^	14 (70)^a^	32 (53.3%)^a^	
**Left TBP**					
Type 1	0a (0%)^a^	0a (0%)^a^	0 (0%)^a^	3 (5%)^a^	**0.001**
Type 2	1 (12.5%)^ab^	0 (0%)^b^	10 (50%)^a^	20 (33.3%)^a^	
Type 3	7 (87.5%)^ab^	20 (100%)^b^	10 (50%)^a^	37 (61.7%)^a^	

*Fisher’s exact test, TBP: temporal bone pneumatization. Column proportions were compared using Z test with Bonferroni correction. The items in parentheses indicate percentages. Same letters (a, b) indicate no significant difference between columns

In terms of the left temporal aeration measurements, one (12.5%) of the skulls obtained from Cyzicus had type 2 pneumatization, and seven (87.5%) had type 3 pneumatization. All the skulls obtained from Belentepe had type 3 pneumatization. While 10 (50%) of the skulls obtained from Karacaahmet were assigned to type 2, 10 (50%) had type 3 pneumatization. Among the contemporary skulls, type 1 was described in three (5%) skulls. We observed that 20 (33.3%) had type 2 pneumatization, and 37 (61.7%) had type 3 pneumatization (*p* = 0.001, Table 1).

In terms of type 2 TBP, no significant differences were present between the Cyzicus and Belentepe skulls, but such pneumatization was statistically significantly lower in the Belentepe skulls than in the Karacaahmet and contemporary skulls. While no significant differences were noted between the Belentepe and Cyzicus skulls in terms of type 3 TBP, type 3 pneumatization was significantly higher in the skulls obtained from Belentepe in comparison to Karacaahmet and contemporary skulls (*p* = 0.001, Table 1).

### Paranasal sinus volumes

The frontal sinus volumes could not be measured in two of the skulls sourced from Cyzicus, and the maxillary and sphenoid sinus volumes could not be measured in one skull. The frontal sinus volume could not be measured in three of the skulls obtained from Belentepe. In the contemporary images, agenesis in the frontal sinus was detected in two skulls. After these had been excluded, no statistically significant differences were found in the PSV (frontal, maxillary, and sphenoid) between the groups (*p* > 0.05, supplementary Table 1).

### Internal acoustic canal and olfactory cleft measurements

When the skulls from the different eras were compared in terms of IAC length, the values for the Karacaahmet skulls on the right and left were found to be statistically significantly lower than those of the contemporary skulls (*p* = 0.006 and *p* = 0.001, respectively, supplementary Table 2). No significant differences were found between the other groups of skulls in terms of right or left IAC length. The IAC width of the contemporary skulls was found to be significantly lower on both sides compared to the skulls from other three eras (*p* < 0.001, supplementary Table 2), and no differences were detected between the latter. The contemporary OC width was found to be statistically significantly lower on both sides when compared to the OC width of the skulls from the other three eras (*p* < 0.001, supplementary Table 2), and no significant differences were found in the comparisons between these three sets of skulls (*p* > 0.05).

## Discussion

In our study, we evaluated the temporal bones and paranasal sinuses pneumatization status of people who lived in societies in Anatolia for periods of approximately 500, 1000, and 2000 years. To the best of our knowledge, no similar study has been published in the literature. Our study showed that while type 3 TBP has decreased over the centuries, type 1 and 2 TBP have increased (Table 1). Accordingly, temporal bone ventilation has decreased over time. Similar to our findings, other studies have suggested that TBP has decreased throughout human evolution, although the significance of this or the process leading to this change is not yet known [10, 20].

We hypothesize that the increases in chronic otitis media with effusion, allergies, and exposure to smoking and an increasing population compared to previous ages may have affected the TBP process. On the other hand, changes in craniofacial dimensions may be another cause of the decrease in TBP, although we did not find any such correlation and have therefore not published these results. It is accepted that reduced mastoid pneumatization is one of the most important factors in the course of adhesive otitis media [21, 22].

Urquiza et al. [1] investigated the temporal bone of *Homo heidelbergensis* and compared their findings with those of other studies. They claimed that a pneumatized temporal bone associated with a more acute canaliculo-fenestral angle, the absence of lesions in the middle ear cavity, and rich pneumatization may indicate that the middle ear is healthy. In addition, Ilea et al. [23] found that mastoid pneumatization plays a role in the absorption and distribution of energy against temporal fracture, which thus reduces the incidence of fracture. Similarly, Kang et al. [20] found that the degree of TBP was negatively correlated with the degree of damage to the otic capsule and hearing loss in patients with temporal trauma. It may therefore be concluded that decreasing TBP over the centuries, which is a finding of our study, makes the individuals more prone to the effects of trauma. In other words, societies that lived centuries ago may have been more resistant to temporal trauma. On the other hand, Balzeau and Grimaud-Hervé [11] and Balzeau and Radovcic [24], who conducted the only studies to date that measured TBP in fossils and nonhuman primates, found that, unlike our study, Neanderthals had a lower mean pneumatization volume and a slightly different pneumatization pattern than modern humans. The researchers deduced that the development of pneumatization in Neanderthals may have continued longer than in modern humans.

We concluded that PSV have not changed over the centuries, and intergroup comparisons of men and woman showed no significant differences. Similarly, Marquez et al. [19] examined the paranasal sinuses of mummies and three different populations that lived in similar regions and found no significant differences. The nose and paranasal sinuses, which have many functions, are affected by genetic factors as well as environmental factors that lead to respiratory adaptation [18]. Marquez et al. [19] found significant differences in the size of the frontal sinuses among different geographical groups with an estimated elapsed time frame of 2,500 to 3,400 years and suggested that environmental conditions may play a more important role in the development of the nose and paranasal sinuses than modern factors. Similarly, we found no paranasal sinus volume changes in the skulls of people who lived in similar climatic areas in different eras, which may support their findings. However, while we did not detect any secular differences between the skulls, Marquez et al. [19] found significant differences in the frontal and maxillary sinus volumes. In another study, Hanson and Owsley [25] investigated sinus size within Canadian Inuit populations, Canadians, American Inuit groups, and Native Americans. Similar to our findings, they found no differences in the frontal sinus size among the Canadian populations and a comparably slightly smaller size among the American Inuit groups. Rae et al. [26] concluded that the climate played a crucial role in nasal cavity and possibly maxillary sinus aeration among Japanese macaques.

In our study, we found that the width and length of the IAC and the width of the OC decreased over the centuries. Considering that the sense of smell and hearing were more vital for life in the studied societies, it could be assumed that they had a better sense of smell and hearing. Another possibility is that these differences may be secondary to differences in TBP and/or craniofacial measurements without affecting end sensory organ sensitivity. de March et al. [27] investigated the in vitro function of 30 odorant receptor genes in the genus *Homo*, and they concluded that it has a shared repertoire with possible local ecological adaptations. In our study, although all the materials were obtained in the lands of Anatolia, geographical and environmental differences may have affected our results. With respect to the IAC, the literature has mainly focused on the role of the angles of the IAC on gender estimation [28], and we could not locate a similar study to ours in the English literature.

It appears a fair inference that changes in size of OC and IAC might be another indication of the fact that olfaction and hearing were more vital for survival in old eras. Since we do not know incidence of chronic ear problems in old eras, we cannot speculate outcome of increased TBP in terms of developing chronic ear diseases. On the contrary, increased TBP was likely to play a protective role in traumas in old ears. Additionally, the authors argue that the environmental influences may be crucial role in the development of paranasal sinuses.

The main limitation of our study was the relatively small number of specimens and unequal gender distribution among ancient skulls. When all the data were analyzed for differences between males and females regardless of skull origin, frontal sinus volumes, maxillary sinus volumes and left olfactory cleft dimensions were statistically higher in males than females. No significant differences were found between the other measurements. Therefore, we believe that this issue should be taken into consideration when evaluating the results of the study. Although all the studied populations had lived in Anatolia, the fact that the skulls were from different regions of Anatolia is another limitation of our study. A strength of our study is that it provides the opportunity to compare four populations that lived approximately 500, 1,000, and 2000 years apart. We believe that future investigations with larger populations that lived in the same or nearby regions with similar genetic characteristics will more clearly reveal the effect of time on the parameters evaluated in our study.

## Conclusion

**A**lthough no significant change was observed in PSV, decreases were evident in TBP, OC width and IAC length and width over time. It appears a fair inference that changes in size of OC and IAC might be another indication of the fact that olfaction and hearing were more vital for survival in old eras. Since we do not know incidence of chronic ear problems in old eras, we cannot speculate outcome of increased TBP in terms of developing chronic ear diseases. On the contrary, increased TBP was likely to play a protective role in traumas in old eras. Additionally, the environmental influences may play crucial role in the development of paranasal sinuses.

## Electronic supplementary material

Below is the link to the electronic supplementary material.

Supplementary Material 1

Supplementary Material 2

Supplementary Material 3

Supplementary Material 4

## Declarations

### Data sharing statement

Data are available or available upon request.
