# Supplementary figures and images for: Changes in paranasal sinus volumes, temporal bone pneumatization, internal acoustic canal and olfactory cleft dimensions over the centuries: a comparison of skulls from different epochs in Anatolia
Source: Eur Arch Otorhinolaryngol. 2024 Jul 8;281(11):5983–90. doi: 10.1007/s00405-024-08804-9 (PMC11512874; doi:10.1007/s00405-024-08804-9)

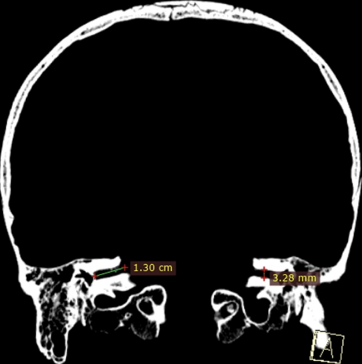


**Supplementary Figure 1.** Measurement of internal acoustic canal length (right) and width (left)

Supplement: Supplementary file 1 — Supplementary Material 1 [file 405_2024_8804_MOESM1_ESM.docx]

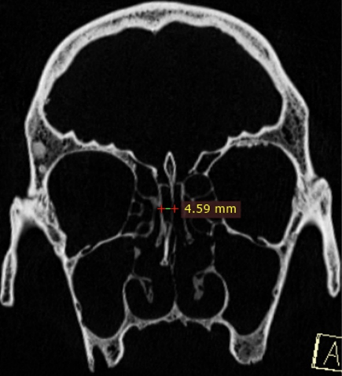


**Supplementary Figure 2.** Measurement of olfactory cleft width.

Supplement: Supplementary file 2 — Supplementary Material 2 [file 405_2024_8804_MOESM2_ESM.docx]
