# Supplementary material for: Changes in paranasal sinus volumes, temporal bone pneumatization, internal acoustic canal and olfactory cleft dimensions over the centuries: a comparison of skulls from different epochs in Anatolia
Source: Eur Arch Otorhinolaryngol. 2024 Jul 8;281(11):5983–90. doi: 10.1007/s00405-024-08804-9 (PMC11512874; doi:10.1007/s00405-024-08804-9)
Supplement: Supplementary file 3 — Supplementary Material 3 [file 405_2024_8804_MOESM3_ESM.docx]

**Supplementary table 1.** Paranasal sinus volume measurements and comparison between groups.

|  | **2^nd^ Century AD** | | | **10^th^-11^th^ Century AD** | | **16^th^-19^th^ Century AD** | | **Contemporary** | |  |  |
| --- | --- | --- | --- | --- | --- | --- | --- | --- | --- | --- | --- |
|  | Mean±SD | | Median (Min-Max) | Mean±SD | Median (Min-Max) | Mean±SD | Median (Min-Max) | Mean±SD | Median (Min-Max) | Test Stats. | p |
| Frontal sinus volume (cm^3^) | | 23.88± 9.94 | 20.74(15.15-41.53) | 17.41±16.08 | 11.74(1.191-67.48) | 25.65±18.18 | 17.65 (8.17-73.09) | 25.89±21.64 | 20.32 (0.362-98.01) | 5.246 | 0.155*** |
| Sphenoid sinus volume (cm^3^) | | 18.97±12.44 | 16.79 (4.1-44.31) | 19.10±9.167 | 15.57 (6.65-41.77) | 20.84 ±10.24 | 21.16 (5.95-42.28) | 22.19±11.71 | 20.58 (3,62-54.14) | 0.500 | 0.683* |
| R maxillary sinus volume (cm^3^) | | 53.37±16.04 | 53.01 (31.54-81.93) | 43.69±14.81 | 44.34 (8.91-72.79) | 40.27 ±11.72 | 41.63 (17.78-63.67) | 40.50±15.51 | 37.20 (5.11-98.23) | 6.156 | 0.104*** |
| L maxillary sinus volume (cm^3^) | | 47.27±16.62 | 42.15 (31.69-78.46) | 43.47±16.09 | 41.01 (14.14-78.09) | 40.98 ±16.26 | 39.75 (10.56-75.31) | 40.60±16.9 | 39.96 (6.19-105.12) | 1.478 | 0.687*** |

*ANOVA, ***Kruskal Wallis, SD: standard deviation, R:right, L: left.
