# Supplementary material for: Changes in paranasal sinus volumes, temporal bone pneumatization, internal acoustic canal and olfactory cleft dimensions over the centuries: a comparison of skulls from different epochs in Anatolia
Source: Eur Arch Otorhinolaryngol. 2024 Jul 8;281(11):5983–90. doi: 10.1007/s00405-024-08804-9 (PMC11512874; doi:10.1007/s00405-024-08804-9)
Supplement: Supplementary file 4 — Supplementary Material 4 [file 405_2024_8804_MOESM4_ESM.docx]

**Supplementary table 2.** Descriptives and comparison of internal acoustic canal width/length and olfactory cleft width.

|  | **2^nd^ Century AD** | | | **10^th^-11^th^ Century AD** | | **16^th^-19^th^ Century AD** | | **Contemporary** | |  |  |
| --- | --- | --- | --- | --- | --- | --- | --- | --- | --- | --- | --- |
|  | **Mean±SD** | | **Median (Min-Max)** | **Mean±SD** | **Median (Min-Max)** | **Mean±SD** | **Median (Min-Max)** | **Mean±SD** | **Median (Min-Max)** | **Test Stats** | **p^Δ^** |
| **Right IAC length (mm)** | | 13.59±1.55^ab^ | 13.22 (11.78-16.48) | 12.52±1.72 ^ab^ | 12,45 (8.46-15.34) | 13.34±2.19 ^a^ | 13.27 (10.22-17.35) | 11.89±1.8^b^ | 11.5 (6.8-16.2) | 4.364 | **0.006*** |
| **Left IAC length (mm)** | | 13.25±1.64 | 13.27 (10.63-16.35)^ab^ | 12.56±1.67 | 12,68 (9.22-15.7)^ab^ | 13.34±1.9 | 13.29 (9.82-17.34)^a^ | 11.74±1.57 | 11.6 (9.2-17.5)^b^ | 16.302 | **0.001***** |
| **Right IAC width (mm)** | | 6.42±1.07 | 6.37 (4.7-8.02)^a^ | 5.46±1.14 | 5,09 (4.46-9.19)^a^ | 5.15±0.93 | 5.14 (3.33-6.86)^a^ | 3.74±0.76 | 3.7 (2.4-6.3)^b^ | 59.370 | **<0.000***** |
| **Left IAC width (mm)** | | 6.06±0.8^a^ | 6,18 (4.73-7.25) | 5.46±0.97^a^ | 5,32 (3.91-7.51) | 5.25±1.16^a^ | 5.13 (3.5-8.09) | 3.86±0.8^b^ | 3.8 (2.4-6.2) | 29.185 | **<0.000*** |
| **Right olfactory cleft width (mm)** | | 3.24±0.49^a^ | 3.34 (2.41-3.79) | 2.77±0.62^a^ | 2,61 (1.9-4.19) | 2.84±0.76^a^ | 2.78 (1.36-4.46) | 2.19±0.44^b^ | 2.25 (1.38-3.21) | 20.569 | **<0.000**** |
| **Left olfactory cleft width (mm)** | | 2.94±0.78 | 2.79 (1.99-3.88)^a^ | 2.84±0.83 | 2,63 (2.06-5.42)^a^ | 3.05±0.67 | 3.15 (1.75-4.22)^a^ | 2.16±0.4 | 2.18 (1.45-3.21)^b^ | 33.915 | **<0.000***** |

*ANOVA, **ANOVA with Welch Correction, ***Kruskal Wallis, IAC: internal acoustic canal, SD: standard deviation. There is no significant difference between groups including the same letter. ^Δ^Mean values for normal distribution and median values for abnormal distribution were used for the statistical analysis.
